# Analyzing the Stressors for Frontline Soldiers Fighting Against Coronavirus Disease 2019 Pandemic

**DOI:** 10.3389/fpsyg.2021.751882

**Published:** 2021-11-18

**Authors:** Muhammad Zeeshan Shaukat, Miklas Scholz, Tehmina Fiaz Qazi, Abdul Aziz Khan Niazi, Abdul Basit, Asif Mahmood

**Affiliations:** ^1^Faculty of Management Studies, University of Central Punjab, Lahore, Pakistan; ^2^Division of Water Resources Engineering, Department of Building and Environmental Technology, Faculty of Engineering, Lund University, Lund, Sweden; ^3^Department of Civil Engineering Science, School of Civil Engineering and the Built Environment, University of Johannesburg, Johannesburg, South Africa; ^4^Department of Town Planning, Engineering Networks and Systems, South Ural State University, Chelyabinsk, Russia; ^5^Institute of Environmental Engineering, Wroclaw University of Environmental and Life Sciences, Wrocław, Poland; ^6^Hailey College of Banking and Finance, University of the Punjab, Lahore, Pakistan; ^7^Institute of Business and Management, University of Engineering and Technology, Lahore, Pakistan; ^8^Lahore Institute of Science and Technology, Lahore, Pakistan; ^9^Department of Business Studies, Namal Institute, Mianwali, Pakistan

**Keywords:** COVID-19, frontline soldiers, healthcare, ISM, MICMAC, coronavirus disease 2019, pandemic and stressors

## Abstract

This study aimed to analyze stressors to which medical staff is vulnerable due to the coronavirus disease 2019 (COVID-19) pandemic. It also imposes a hierarchy on complex relations among stressors for excavating underlying structure and builds a model of interrelationships contrasting reality. The design of this study comprises a literature survey, data collection from primary sources, and analysis. Stressors have been explored from within current published/unpublished literature and validated by experts through approval vote. Data were collected from the focus group (panel of experts), and interpretive structural modeling (ISM) was used as the research methodology. Findings of ISM are avowed through “cross-impact matrix multiplication applied to classification” (MICMAC) analysis. As a result of the literature survey, a list of stressors was generated, and a total of 19 stressors qualified as representative of the phenomenon. The results of ISM show that two stressors (i.e., “unavailability of proper personal protective equipment (PPE)” and “lack of proper communication”) emerged as the most critical stressors since they occupy the bottom of the model, whereas, four stressors (i.e., “anxious about isolation/quarantine,” “subject to violent crimes,” “feeling frustrated and powerless,” and “exhausting shifts/hours without clear end”) are relatively less critical since they occupy the top of the model. The rest of the stressors occupy the middle of the model and therefore, have moderate-severe effects on frontline soldiers. The results of MICMAC show that the stressor “subject to violent crimes” is classified in the dependent cluster and the remaining fall in the linkage cluster but no stressor falls in independent and autonomous. Overall results indicate that all stressors are relevant to the phenomenon under this study, but they are currently not settled. This study is invaluable for policymakers, frontline soldiers, researchers, the international community, and society since it provides a lot of new information that is helpful in refining strategies and combating influential stressors.

## Introduction

With the outbreak of the coronavirus disease 2019 (COVID-19) pandemic in 2019 from Wuhan, China, the dynamics of healthcare changed altogether. The frontline medical staff (i.e., doctors, nurses, and paramedical staff) and their families are exposed to life threats during the pandemic. Some of them even lost their lives and others passed through shocks. COVID-19 outbreak was reported in a small number of countries till March 2020, including China, South Korea, Iran, and Italy, while many more had seen the calm before the storm (Finset et al., 2020). Coronavirus disease is wreaking havoc across civilization, including mental and physical health. COVID-19 has put extra demand on hospital resources and thrown contemporary healthcare procedures into disarray across the world. Focus shifted from quality treatment with ample resources to distributing scarce resources among patients in an equitable manner. Even the most advanced nations with the best healthcare systems are unable to cope with an unexpected rise in the number of patients requiring treatment, particularly in intensive care and mechanical breathing (Smereka and Szarpak, 2020). The number of patients requiring hospitalization and critical care assistance has rapidly increased (Dargaville et al., 2020; Tuite et al., 2021). Overcrowding, lack of isolation rooms, and environmental pollution exacerbated disease transmission (Zhang et al., 2020). Current predictions imply an insufficient supply of personal protective equipment (PPE) to protect physicians at risk due to growing demand worldwide (Binkley and Kemp, 2020). Healthcare professionals are confronting a large volume of seriously sick patients requiring advanced life-sustaining treatments in intensive care units (ICUs) (Akgün et al., 2020). Increased workload and life-threatening situations that medical personnel face during the COVID-19 pandemic have engendered psychological stress leading to mental illness (Lu et al., 2020).

Researchers and clinicians are interested in evaluating and analyzing the nature and magnitude of response to acute psychological stress. In this context, researchers worldwide emphasized devoting time and resources to study psychological stress for characterizing better responses (Turner et al., 2020; Liu Y. et al., 2021). Medical personnel and their families are undoubtedly under pressure owing to the threat of infection and are susceptible to COVID-19 transmission. In reality, the entire health system, which involves a variety of stakeholders, is under strain. Stakeholders of health systems [e.g., medical personnel (i.e., physicians, nurses, paramedics, and technicians working in radiology, labs, theaters, dialysis units, etc.), hospital board of trustees, management, professional and non-professional, suppliers, patients, the financial community, competitors, government regulatory agencies, private accrediting bodies, professional associations, labor unions, and media and political action groups] are anxious about the unsettled vista of current health systems (Pereno and Eriksson, 2020). The pandemic counter generated a range of stressors for frontline soldiers. Thus, it has become imperative to specify, evaluate, and analyze current uncharacterized stressors faced by the frontline soldiers.

Against this backdrop, this research is conducted from the perspective of one of the most important stakeholders, i.e., medical staff. Objectives of the study are to (i) identify stressors for frontline soldiers, (ii) determine contextual relationships among them, (iii) build a structural model underlying the contextual relationships of the stressors, and (iv) discuss how the model is helpful for policymakers and other stakeholders. Interpretive structural modeling (ISM) with cross-impact matrix multiplication applied to classification (MICMAC) (Warfield, 1973, 1974; Sushil, 2017) is opted as a research methodology to achieve the objectives. It is a theory-building mathematical technique that outperforms its statistical rivals. This study is divided into five sections, namely, introduction, literature review, methodology, data collection, analysis results and discussion, and conclusion.

## Literature Review

Since literature provides hard ground to start with, averts duplication, helps refine the research gap, and aids in acknowledging this research, we explored contemporary literature thoroughly. Since stress is a new phenomenon in the scenario of the COVID-19 pandemic situation, this literature, whether published, accepted for publication, or unpublished, has been targeted for review. We used Google as a search engine and explored research databases of SpringerLink, Emerald, JSTOR, Wiley-Blackwell, Elsevier (Science Direct), and Taylor & Francis. A number of studies were reviewed, but in this study, only the findings of some relevant studies are reported for brevity.

Coronavirus disease 2019 poses a significant public health threat worldwide. It is a complex, contagious, and typically vulnerable epidemic (Rutherford et al., 2021). It causes significant problems in terms of societal prevention and control (Mo et al., 2020). Lu et al. (2020) argued that fear, worry, and sadness are more prevalent among the medical personnel than among the administrative staff during the COVID-19 pandemic. They also argued that frontline medical professionals in respiratory, emergency, ICU, and infectious disease departments are twice as likely to experience anxiety and sadness than non-clinical employees who had less interaction with patients with coronavirus pneumonia. Of note, 42% of doctors working at tertiary hospitals in mainland China had extremely high levels of accumulated tiredness (Tang et al., 2019; Ardebili et al., 2021). Healthcare workers require sufficient protection and training to use equipment in order to provide safe care. However, when hospitals fail to provide appropriate PPE, it is impossible to provide safe healthcare. This creates a sense of inadequacy and undervaluation among patient-focused healthcare workers, resulting in workplace stress (Herron et al., 2020). Taylor et al. (2020) introduced a five-factor assessment scale for measuring COVID-19 stress and anxiety, which includes a scale for (i) COVID danger and contamination, (ii) COVID socioeconomic consequences, (iii) COVID xenophobia, (iv) COVID traumatic stress symptoms, and (v) COVID compulsive checking. During the COVID-19 epidemic, medical staff, particularly those in the Wuhan area, experienced more psychological stress than college students, demonstrating an evident “exposure effect” (Wu W. et al., 2020). Holmes et al. (2020) investigated the psychological, sociological, and neuroscientific effects of COVID-19. They also investigated immediate and long-term research goals in the context of mental health science. Grace and VanHeuvelen (2019) found that burnout in doctors and nursing professionals is significantly greater than their lower-status counterparts, and much of this may be ascribed to job pressures reported by higher-status practitioners. Nurses are continuously under pressure while facing the current pandemic (Sun et al., 2020).

Similarly, COVID-19 has a significant influence on the everyday practice and surgical education of a surgeon. Elective and non-urgent surgical cancelations have made doctors a valuable resource for health systems dealing with the COVID-19 epidemic. Surgeons are examining safe, non-surgical alternatives to treat their patients (Al-Jabir et al., 2020). Medical professionals operating on the frontlines showed a lower burnout rate than those working in their normal ward for uninfected patients. Therefore, it will be critical to include both frontline healthcare professionals and those in their regular work settings (Wu Y. et al., 2020). Reznik et al. (2020) formulated some useful findings that might be helpful for online training of trainers, who could then work with others to help avoid or minimize COVID-19-related fear, stress, and anxiety because these factors can lead to the use of the hazardous substance, domestic violence, or criminality.

Gritsenko et al. (2020) concluded that pandemics such as COVID-19 cause psychological stress, emotional disturbance, sadness, irritation, sleeplessness, rage, emotional weariness, and other physiological and mental health issues. Shen et al. (2020) suggested reducing the psychological strain on ICU nurses quickly. Liu C. H. et al. (2021) argued that in order to reduce the mental health burden of COVID-19, researchers must first identify the risk factors for post-traumatic stress disorder (PTSD) and persistent psychological discomfort. North et al. (2021) asserted that the COVID-19 pandemic is unique in numerous aspects, and the mental health concerns connected with the pandemic are unknown. They also stressed that there is a severe need to investigate COVID-19 data on socio-demographic variables, exposure-related factors (e.g., living in highly affected areas, knowing or having a close relationship with someone infected with COVID-19, becoming infected with COVID-19, being quarantined or hospitalized for COVID-19, and working on the frontline of the COVID-19 pandemic), effects of loss of beloved ones, and pandemic-related worries and stressors. Furthermore, fear of being infected, concerns about health and safety, financial losses, job loss, housing problems, social isolation, and lack of support of family members have increased the risk of PTSD and chronic psychological distress (Boyraz and Legros, 2020; North et al., 2021).

It is also important to note that worry and anxiety are natural responses to dangerous and unpredictable situations. Likewise, unsteady focus, irritation, anxiety, sleeplessness, lower productivity, and interpersonal problems are all possible stress-related behaviors in response to the coronavirus pandemic (Vinkers et al., 2020). Taylor et al. (2020) argued that many people display fear and anxiety-related distress reactions during the pandemic, which include dread of contracting an infection, fear of coming into touch with potentially contaminated things or surfaces, fear of infection from foreigners (i.e., disease-related xenophobia), fear of the socioeconomic consequences of the pandemic (e.g., job loss), compulsive checking and reassurance-seeking regarding possible pandemic-related threats, and traumatic stress symptoms about the pandemic (e.g., nightmares and intrusive thoughts).

Despite these investigations, further studies on the psychological experiences of frontline medical staff combating novel COVID-19 would be helpful to make some sense of possible solutions (Sun et al., 2020). Currently, there have been fewer studies comparing the mental health of healthcare professionals across occupational categories or determining the extent to which occupational diversity in mental health among these employees may be related to the diverse work circumstances they experience (Grace and VanHeuvelen, 2019; Sovold et al., 2021). Specifically, understanding the psychological effects of the COVID-19 pandemic on healthcare professionals (as frontline soldiers) is critical for formulating progressive policies (Tan et al., 2020; Liu C. H. et al., 2021).

Thus, extending the research in this direction, a list of stressors apropos to the literature review has been generated in Table 1.

**TABLE 1:** Stressors for frontline soldiers fighting due to coronavirus disease 2019 (COVID-19) pandemic.

Code	Stressor	Description	Source
1	High job demands	Job demands may be physical, psychological, social, or organizational that require continuous physical and/or psychological (i.e. cognitive or emotional) effort.	Grace and VanHeuvelen, 2019
2	Low job control	When workers have less ability to make decisions about the way they work or use their skills.	Grace and VanHeuvelen, 2019
3	Risk of COVID-19 infection	Fear of self-infection while treating the COVID-19 patients	Zhang et al., 2020
4	Unavailability of proper PPEs	Non-availability of personal protective equipment (PPEs) because there is a shortage worldwide, and it is also not reaching the concerned staff in Pakistan.	Binkley and Kemp, 2020
5	Inadequate training	Since the pandemic broke out suddenly, medical staff is neither already trained nor can be trained in panic.	Wu Y. et al., 2020
6	Work-family conflict	Work-family conflict occurs when individual experiences incompatible demands between work and family roles; participating in both roles at a time becomes difficult.	Lu et al., 2020
7	Anxious about isolation/quarantine	Anxious about isolation or quarantine in case of any eventual chance of COVID-19 infection.	Boyraz and Legros, 2020
8	Subject to violent crimes	There are certain instances of violent crimes by COVID-19 patients/suspected patients or by immediate attendants of patients.	Reznik et al., 2020
9	Feeling frustrated and powerless	Workers’ state is helpless among so many patients of COVID-19.	Herron et al., 2020
10	Worried on magnitude of problem	Workers are worried about massive spread and proliferation without any definite end.	Boyraz and Legros, 2020
11	Exhausting shifts/hours without a clear end	Exhausting hours without a clear end.	Gritsenko et al., 2020
12	Shell shock	Workers’ state of involuntarily shivering, crying or fear, and had constant intrusions of memory.	Taylor et al., 2020
13	Post-traumatic stress disorder (PTSD)	A mental health condition triggered by a terrifying event like COVID-19 while experiencing or witnessing it, e.g. severe anxiety, uncontrollable thoughts about the event, etc.	Holmes et al., 2020; Greene et al., 2021
14	Fear from co-workers being COVID-19 positive	Workers’ state of being uncertain about the co-workers that they might be COVID-19 positive.	Taylor et al., 2020
15	No mental work break	Working for long stretches without breaks lead to stress and exhaustion.	Lu et al., 2020
16	Lack of proper communication	Lack of proper strategic communication by health department/regulators.	Vinkers et al., 2020
17	Afraid of spread of COVID-19 in own family	Afraid of the spread of COVID-19 in own family/friends/relatives.	Boyraz and Legros, 2020
18	Sad for too many dyeing patients	Workers’ state of sadness on numerous deaths due to COVID-19 world over.	Suggested by experts
19	Shortage of ventilators, beds, etc.	Shortage of necessary lifesaving equipment like ventilators, beds, testing kits, etc.	Dargaville et al., 2020

Initially, a list of 18 stressors was generated from literature; the same was presented to the experts for ratification through approval vote. The experts had the option to exclude, merge, and/or add stressors on the basis of their expert knowledge. All the factors extracted from the literature were approved; one stressor was added by experts as listed in serial no. 18 in Table 1. Since a multitude of 16–20 elements (stressors in this study) suffices to develop an understanding of contextual relationships of the phenomenon using ISM (Sushil, 2017), the study proceeded accordingly.

## Methodology

This study follows positivist research philosophy and induction as a research approach. The design of the study includes a review of contemporary literature, collection of primary data by field survey, and analysis. This section is divided into two subsections: population, sampling, and procedure and techniques used.

### Population, Sampling, and Procedure

The population under investigation is the medical staff (i.e., physicians/doctors, nurses, paramedics, and technicians working in radiology, labs, theaters, dialysis units, and other areas). This study uses a non-probability-based purposive sampling technique (Ranjbar et al., 2012) to elicit data on 171 direct (*ij*) paired relations from a panel of experts using face-to-face interviews (Li and Yang, 2014). There are two options for recruiting such groups, namely, homogeneous and heterogeneous, each requiring different group sizes. The panel size for homogeneous groups varies from 12 to 25 experts and that of heterogeneous from 8 to 16 experts (Clayton, 1997; Khan and Khan, 2013). In this study, we opted for a homogeneous group of frontline medical staff with a panel size of 18 experts. However, we recruited a heterogeneous group from within the medical staff. The panel was recruited based on their practical, theoretical, and expert knowledge about the phenomenon under study and relevant experience in an authoritative organization. The principle followed in recruiting the panel experts was “*quality is more important than quantity*” (Clayton, 1997; Shen et al., 2016). The piloting function was performed before the data collection through pretesting, emails, phone calls, and personal meetings to invite experts to participate in this study. It took almost 4 months to complete the process of data collection. More than 50 experts were approached, only 20 agreed to participate, but 18 actually participated. The participating panel consisted of one researcher, six physicians/doctors, four nurses, four paramedics, two radiologists, and one lab staff. All the participants were university graduates in the relevant field, having a minimum experience of 10 years in reputed organizations, and are deputed on the frontline to fight against COVID-19. The panel was approached three times: first, for obtaining their judgment as to whether the stressors are reasonable, representative, and sufficient to capture the phenomenon; second, for data collection regarding interrelationships of stressors; and third, for reviewing logical, theoretical, conceptual, and directional consistency of the model (Raeesi et al., 2013; Vasanthakumar et al., 2016).

### Techniques Used

Four techniques have been used in this study for different purposes as detailed below.

### The Technique Used for Identification of Factors

Several methods are used for the identification of factors: opinion of experts (Avinash et al., 2018; Niazi et al., 2019a); literature review (Ali et al., 2018); Delphi method (Bhosale and Kant, 2016); case study (Li et al., 2019); exploratory factor analysis (Li and Yang, 2014); meta-analysis (Lohaus and Habermann, 2019); presumed by authors (Lohaus and Habermann, 2019); interview content analysis (Xiao, 2018); idea engineering and brainstorming (Kumar et al., 2013); empirical evidence provided, anecdotal evidence from literature, and literature review based on purposive sample (Azevedo et al., 2013). In this study, a literature review combined with the approval of experts has been used to finalize the list of stressors. The discourse of literature was opted because literature provides hard ground/current knowledge of the topic, prevents objectionable duplication of work, pinpoints questions open to research, and helps justify the contribution of the study to existing literature with due credit to other researchers. Then, the data were collected on *ij* part of the matrix, i.e., evaluation of every paired relation by every expert on the panel (Alawamleh and Popplewell, 2011; Trigunarsyah and Parami Dewi, 2015).

### The Technique Used for Eliciting Data From the Panel of Experts

There are numerous methods to elicit data from a panel of experts, e.g., Delphi method, brainstorming session, discussion session, nominal group technique (NGT), repertory-grid interview technique (RGT), matrix type questionnaire, laddering interview, problem-solving group session, in-depth discussion, face-to-face in-depth interview, triadic sorting task approach, approval voting on alternatives (VAXO) for every pair of relations through software/questionnaire, workshop, and idea engineering or idea generation method with small group exercise (Baruah and Paulus, 2008; Niazi et al., 2020, 2019b). This study uses face-to-face approval VAXO for every pair of relations through matrix type (i.e., (*n*(*n*−1))/2 matrix) questionnaire to capture the mental model of the expert without loss of original data (Supplementary Annexure 2). It is a conscious decision to get the *ij* part of the questionnaire filled by the respondent to avoid the subjective involvement of researchers.

### The Technique Used for Model Development

To develop the model, we considered an array of methodologies (Shaukat et al., 2021) and found ISM with MICMAC as the most appropriate technique since it is a simple one to serve the purpose objectives like this study (Warfield, 1973, 1974; Sushil, 2017). ISM is a methodology that has the capability to transform unclear and poorly articulated mental models of systems into visible, well-defined useful models (Sushil, 2012). It is a mathematical modeling methodology that can simplify complex phenomena (such as understudy) through the permutation of binary matrices. ISM with MICMAC (Matrice d’Impacts Croises Multiplication Appliquée a un Classement) is used as research methodology and modeling to clarify the dependence-independence power of the variables involved.

### The Technique Used for Classification of Stressors and Analyzing Driving Dependence

Cross-impact matrix multiplication applied to classification analysis (Godet, 1986) is applied to classify the stressors into clusters to understand the structure more clearly and analyze the driving and dependence of stressors. It is a structural analysis technique based on the multiplication properties of binary matrices.

## Analysis, Results, and Discussion

### Analysis

There are two parts of analysis, namely, ISM and MICMAC. The analysis starts with applying ISM to the data collected. ISM proceeds stepwise as depicted in Supplementary Figure A1 in Supplementary Annexure 1. Structural self-interaction matrix (SSIM) was prepared by aggregating the data collected from experts through *ij* part of the questionnaire. The data were aggregated by applying the rule “minority gives way to the majority” on every paired relation (Sushil, 2012; Abdullah and Siraj, 2014; Dhochak and Sharma, 2016; Cai and Xia, 2018; Li et al., 2019), as shown in Table 2.

**TABLE 2:** Structural self-interaction matrix (SSIM).

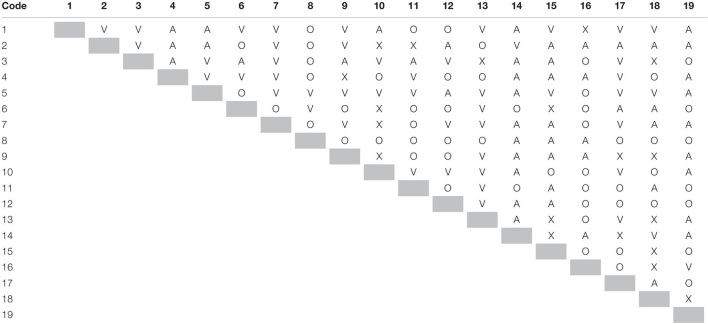

*Shaded diagonal cells are ii part of the matrix that differentiates ij part of the matrix from ji.*

Then, SSIM converted into an initial reachability matrix (Table 3), applying the rules devised by Warfield (1973) and iterated by Attri et al. (2013) and Thakkar et al. (2008).

**TABLE 3:** Initial reachability matrix.

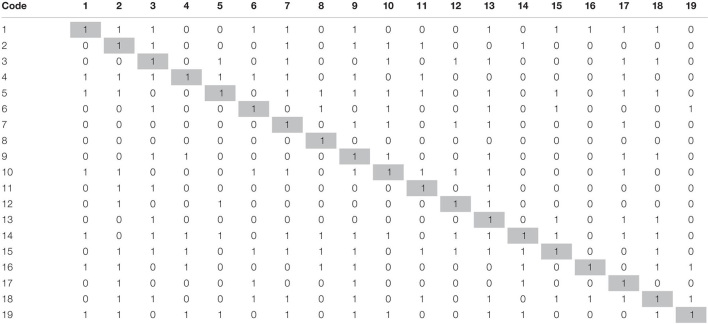

*Shaded diagonal cells are ii part of the matrix that differentiates ij part of the matrix from ji.*

Then, initial reachability was converted to the final reachability matrix by ascertaining the possibility of converting every 0 into 1 due to transitive relation. This check is performed using some MS Excel functions and prepared a fully transitive final reachability matrix. The transitive relations have been distinguished with 1* in the final reachability matrix (Table 4).

**TABLE 4:** Final reachability matrix.

Code	1	2	3	4	5	6	7	8	9	10	11	12	13	14	15	16	17	18	19	Driving
1	1	1	1	1*	1*	1	1	1*	1	1*	1*	1*	1	1*	1	1	1	1	1*	19
2	1*	1	1	1*	1*	1*	1	1*	1	1	1	1*	1*	1	1*	0	1*	1*	0	17
3	1*	1*	1	0	1	1*	1	1*	1*	1	1*	1	1	1*	1*	1*	1	1	1*	18
4	1	1	1	1	1	1	1	1*	1	1*	1	1*	1*	1*	1*	1*	1	1*	1*	19
5	1	1	1*	1*	1	1*	1	1	1	1	1	1*	1	1*	1	1*	1	1	1*	19
6	1*	1*	1	1*	1*	1	1*	1	1*	1	1*	1*	1	1*	1	0	1*	1*	1	18
7	1*	1*	1*	1*	1*	1*	1	0	1	1	1*	1	1	1*	1*	0	1	1*	0	16
8	0	0	0	0	0	0	0	1	0	0	0	0	0	0	0	0	0	0	0	1
9	1*	1*	1	1	1*	1*	1*	0	1	1	1*	1*	1	1*	1*	1*	1	1	1*	18
10	1	1	1*	1*	1*	1	1	1*	1	1	1	1	1	1*	1*	1*	1	1*	1*	19
11	0	1	1	0	1*	0	1*	0	1*	1*	1	1*	1	1*	1*	0	1*	1*	0	13
12	1*	1	1*	0	1	0	1*	1*	1*	1*	1*	1	1	1*	1*	0	1*	1*	0	15
13	0	1*	1	1*	1*	1*	1*	1*	1*	1*	1*	1*	1	1*	1	1*	1	1	1*	18
14	1	1*	1	1	1	1*	1	1	1	1	1*	1	1	1	1	1*	1	1	1*	19
15	1*	1	1	1	1*	1	1	1	1	1*	1	1	1	1	1	1*	1*	1	1*	19
16	1	1	1*	1	1*	1*	1*	1	1	1*	1*	1*	1*	1	1*	1	1*	1	1	19
17	1*	1	1*	1*	1*	1	1*	1*	1	1*	1*	1*	1*	1	1*	0	1	1*	1*	18
18	1*	1	1	1*	1*	1	1	1*	1	1*	1	1*	1	1*	1	1	1	1	1	19
19	1	1	1*	1	1	1*	1	1*	1	1	1*	1*	1	1	1*	1*	1*	1	1	19
Dependence	16	18	18	15	18	16	18	16	18	18	18	18	18	18	18	12	18	18	14	323

The transitive relations have been distinguished with 1* in the final reachability matrix.

**FIGURE 1:**
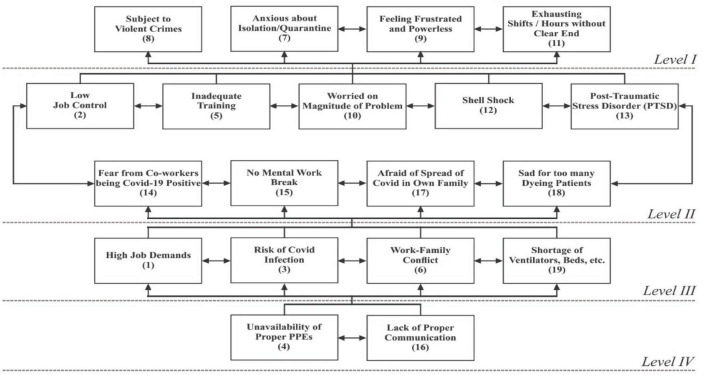
Interpretive structural model.

Subsequently, the final reachability matrix was partitioned into hierarchies (Table 5 and Figure 1) using the iteration method (including conical matrix and digraph) (Warfield, 1973; Sushil, 2012), as shown in Supplementary Tables A1–A6 of Supplementary Annexure 3.

**TABLE 5:** Abridged representation of interpretive structural modeling.

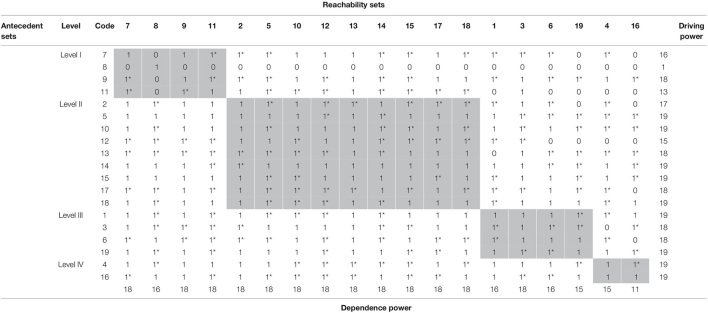

The transitive relations have been distinguished with 1* in the final reachability matrix. Shaded diagonal cells are ii part of the matrix that differentiates ij part of the matrix from ji.

**FIGURE 2:**
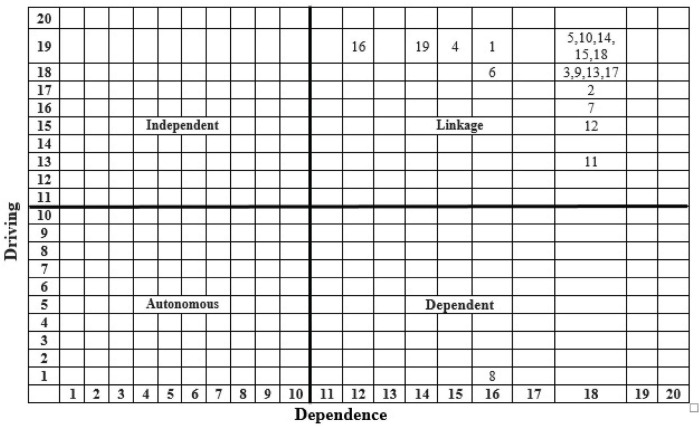
Driving-dependence diagram.

As a result of partitioning, stressors 7, 8, 9, and 11 occupy the top level (*Level I*) of the ISM model; stressors 2, 5, 10, 12, 13, 14, 15, 17, and 18 were placed in the upper-middle level (*Level II*); stressors 1, 3, 6, and 19 occupy lower middle (*Level III*), and stressors 4 and 16 were positioned at the bottom of ISM model (*Level IV*). The MICMAC analysis was applied to classify the stressors into clusters to understand the structure more clearly (Godet, 1986), as depicted in Figure 2.

The results of MICMAC show that all stressors (1, 2, 3, 4, 5, 6, 7, 9, 10, 11, 12, 13, 14, 15, 16, 17, 18, and 19) except “subject to violent crimes” (8) are classified in linkage cluster, meaning thereby agile and ambivalent. Factor, “subject to violent crimes,” is classified as dependent, while none of the stressors is classified as autonomous.

### Results

In the COVID-19 pandemic frontline, medical staff is exposed to life threats. They and their families are vulnerable in the severely affected time of the pandemic. It is imperative to rethink and evaluate stressors faced by frontline soldiers. Therefore, this study aims to prepare a list of stressors, evaluate, analyze, and understand their complex relationships. The literature survey results show 19 critical stressors (Table 1) contributing to the phenomenon under investigation. The results of ISM show that the following stressors occupy top level (*Level I*): anxious about isolation/quarantine (7), subject to violent crimes (8), feeling frustrated and powerless (9), and exhausting shifts/hours without clear end (11). Similarly, the following stressors gained upper-middle level (*Level II*): low job control (2), inadequate training (5), worried about the magnitude of the problem (10), shell shock (12), post-traumatic stress disorder (PTSD) (13), fear from coworkers being COVID-19 positive (14), no mental work break (15), afraid of the spread of COVID in own family (17), and sad for too many dying patients (18), whereas, the stressors that occupy the lower middle level (*Level III*) are as follows: high job demands (1), risk of COVID infection (3), work-family conflict (6), and shortage of ventilators, beds, etc. (19). Furthermore, the following two stressors were placed at the bottom of the model (*Level IV*): unavailability of proper PPEs (4) and lack of proper communication (16). Then, the MICMAC analysis categorized the stressors into the following four clusters (Figure 2): *autonomous cluster:* factors with low driving and dependence powers, separated from the model, have no substantial impact on the system. There is no stressor classified in the autonomous cluster. It implies that all stressors are relevant to the phenomenon under study. *Dependent cluster:* the factors found in this cluster have weak driving but strong dependence, and resultantly, they depend on others. The stressor “subject to violent crimes (8)” is placed in this cluster. *Linkage cluster:* the factors that fall in this cluster have strong driving power and strong dependence power. These factors are agile and unbalanced, and any action on them affects other factors with also a feedback effect on them. The following stressors were observed in this cluster: high job demands (1), low job control (2), risk of COVID infection (3), unavailability of proper PPEs (4), inadequate training (5), work-family conflict (6), anxious about isolation/quarantine (7), feeling frustrated and powerless (9), worried on the magnitude of the problem (10), exhausting shifts/hours without clear end (11), shell shock (12), post-traumatic stress disorder (PTSD) (13), fear from coworkers being COVID-19 positive (14), no mental work break (15), lack of proper communication (16), afraid of the spread of COVID in own family (17), sad for too many dying patients (18), and shortage of ventilators, beds, etc. (19). Since most of the factors fall in the linkage cluster, it implies that the system under study is in its infancy. However, the regulators are struggling to make some sense. *Independent cluster:* the factors that fall in this cluster have high driving but low dependence power. These are key factors that need extra care from regulators. There is no stressor classified as independent, but 13 stressors (Figure 2) have very high driving power but also have high dependence at the same time (classified as linkage). These stressors can be considered as potential independent factors. The results mentioned above have been juxtaposed for convenience in Table 6.

**TABLE 6:** Juxtaposed results of cross-impact matrix multiplication applied to classification (MICMAC) and interpretive structural modeling (ISM).

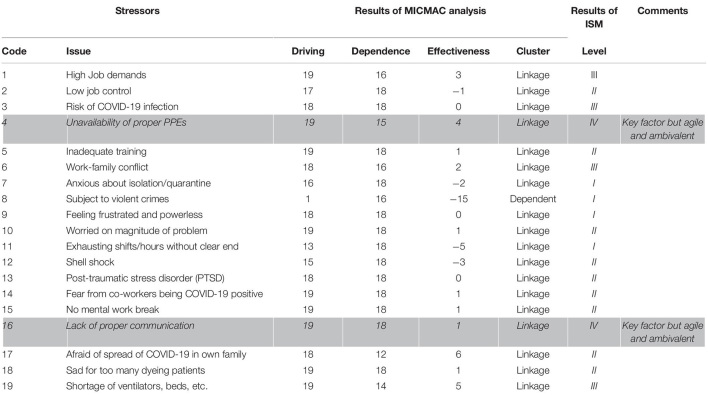

*Shaded diagonal cells are ii part of the matrix that differentiates ij part of the matrix from ji. Stressor marked as grey shaded and italicized are key stressors.*

### Discussion

An influx of studies on stress has been conducted on medical staff due to its frontline fighting against the COVID-19 pandemic. The studies have adopted different discourses to address the issue that varies from revisiting the literature of workplace stressors to identifying new novel stressors. In this regard, the topics of interest include revising scales of measurement, regional, country, or block level comparison. Most of the studies use standard classical statistical methods that utilize massive data but produce meager results. In contrast, this study is exploratory in nature, uses an interpretive paradigm, and employs purposive sampling to produce meaningful and valuable results. This study has more depth than breadth that distinguishes it from contemporary literature. It is different in methodology, dataset, number of variables, results, and depth of investigation. The context of the study (i.e., COVID-19 pandemic situation) is also a distinguishing feature of this study. However, some of the existing studies are conceptually comparable with this study; therefore, a contrast given in Table 7 would help understand the study findings.

**TABLE 7:** Comparison of results of this study with prior studies in the literature.

Sr.	Study	Country	Focus	Method	Results
1	Current	Pakistan	Stressors for frontline soldiers fighting against COVID-19 pandemic	ISM and MICMAC	“Unavailability of proper PPEs” and “Lack of Proper Communication” are the key variables.
2	Vinkers et al., 2020	Netherlands	Stress resilience during the coronavirus pandemic	Consensus statement	Urgent need of strategies to enhance resilience.
3	Grace and VanHeuvelen, 2019	United States	Burnout among medical staff	Classical, traditional statistics	Physicians and nurse practitioners report more work-life conflict, irregular work hours and heavy work pressure than their other colleagues.
4	Taylor et al., 2020	United States/Canada	Development and initial validation of the COVID Stress Scales	Factor analysis (exploratory and confirmatory)	Developed the 36-item COVID Stress Scales (CSS) to measure these features, as they pertain to COVID-19.
5	Gritsenko et al., 2020	Russia/Belarus	COVID-19 fear, stress, anxiety, and substance	Classical elementary statistics with *t*-test and χ^2^ test	Overall, students from Belarus report more positive psycho-emotional conditions and less substance use than those from Russia.
6	Mo et al., 2020	China	Work stress among Chinese nurses	Descriptive single factor correlation and multiple regression analyses	Nurses who fight against COVID-19 are under high pressure.

## Conclusion

With the outbreak of the COVID-19 pandemic in 2019 from Wuhan, China, the dynamics of healthcare have changed altogether. The frontline medical staff (i.e., doctors, nurses, and paramedical staff) and their families are continuously exposed to life threats during the severely affected time of the pandemic. It is also important to ensure that policies and practices are in place to minimize their exposure to respiratory pathogens, including COVID-19. This study on medical staff has explicated the stressors, hierarchized, and simplified them by extracting a structural model underlying the complex relations of stressors. This study uses the discourse of literature review, ISM, and MICMAC as the research methodology. The literature survey results indicate 19 critical stressors (Table 1) contributing to the phenomenon under investigation. The results of ISM show that two stressors, namely, “unavailability of proper PPEs” (4) and “lack of proper communication” (16) have emerged as the most important factors. Since these two factors would have a domino effect on all other stressors, hospital management should put their efforts into minimizing them. The results of this study are consistent with the previous findings (Taylor et al., 2020; Vinkers et al., 2020).

### Contribution of This Study

It has valued theoretical contributions such as identification of stressors for frontline soldiers fighting against COVID-19 pandemic, ISM model, driving-dependence (MICMAC) diagram, condensed demonstration of complex relationships among stressors and auxiliary information in the form of abridged results, comparison of the results of the study with existing studies, and discussion.

### Practical and Theoretical Implications

This study has noteworthy theoretical, practical, policy, and social implications. It is theory-building research and contributes a theoretical framework for designing future investigations. It is helpful for medical staff (i.e., physicians, nurses, paramedics, and technicians working in radiology, labs, theaters, dialysis units, and in other areas), non-medical hospital staff, and their families since it provides a lot of first-hand empirical information regarding the current stressors for them. It will be helpful for them to take informed reposition to become new normal. Patients and their families can take advantage of the study by understanding the current position of medical staff and their families. Suppliers, the financial community, and competitors can benefit from the study by understanding current stressors, their complex relations and hierarchy, readjusting their positions, and revisiting their policies. This study is helpful to the hospital board of trustees/management, government regulatory agencies, accreditation agencies, and professional associations in policymaking since it identified a multitude of stressors built by the COVID-19 pandemic on medical and non-medical hospital staff. It also simplified inter-stressor relationships and found that the system is not settled as yet. It also indicated the driving-dependence power of stressors. This study also prioritized and hierarchized the stressors for setting priorities in policymaking. Medical/non-medical staff unions, media, and political action groups can benefit from the study by understanding current stressors, their complex relations, and hierarchy. Since this study has contributed a theoretical model, driving-dependence diagram, and a lot of information on the phenomenon, it provides a framework for future research.

### Limitations of This Study and Directions for Future Research

First, since the scope and generalizability of research is the limited utility of ISM as a method, future studies should use other suitable multi-criteria decision-making techniques. Second, ISM is based on limited data that only explains what is related to what and how; therefore, it is recommended that future studies should use TISM, fuzzy-ISM, modified-TISM, polarized-TISM, or SEM to quantify causal relationships. Third, since the key stressors have been identified from the literature and/or the judgment of experts, some bias or some factors might have been overlooked. Therefore, it is recommended that future researchers must use other methods to generate an exhaustive list of stressors. Fourth, the research has been conducted in Pakistan, but since there are varying cultural, social, technological, and political systems, generalization of results is limited. Therefore, future studies may be designed in different settings to enhance the frontiers of study.

## Data Availability Statement

The original contributions presented in the study are included in the article/Supplementary Material, further inquiries can be directed to the corresponding authors.

## Ethics Statement

Ethical review and approval was not required for the study on human participants in accordance with the local legislation and institutional requirements. The participants provided written information/response with free consent to participate in this study.

## Author Contributions

MSh generated the idea and worked on introduction and analyses. AN refined the idea and worked on methodology. TQ worked on literature and methodology. AB completed the write up. AM worked on literature. MSc contributed to funding acquisition, supervision of research, and writing – review and editing. All authors contributed to the article and approved the submitted version.

## Conflict of Interest

The authors declare that the research was conducted in the absence of any commercial or financial relationships that could be construed as a potential conflict of interest.

## Publisher’s Note

All claims expressed in this article are solely those of the authors and do not necessarily represent those of their affiliated organizations, or those of the publisher, the editors and the reviewers. Any product that may be evaluated in this article, or claim that may be made by its manufacturer, is not guaranteed or endorsed by the publisher.
