# Supplementary material for: Analyzing the Stressors for Frontline Soldiers Fighting Against Coronavirus Disease 2019 Pandemic
Source: Front Psychol. 2021 Nov 18;12:751882. doi: 10.3389/fpsyg.2021.751882 (PMC8636429; doi:10.3389/fpsyg.2021.751882)
Supplement: Supplementary file 1 [file Data_Sheet_1.pdf]

Annexure 1

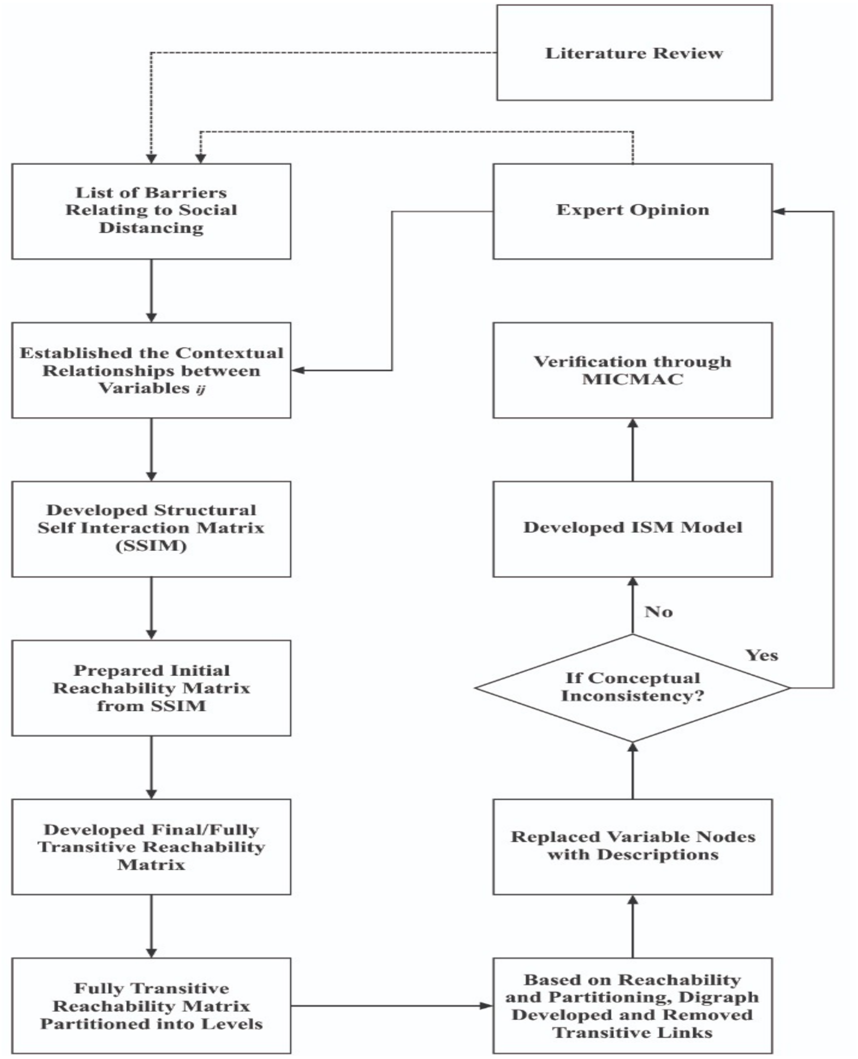

Figure A1: Flow Chart to Apply ISM

Annexure 2

Summarized Research Questionnaire

Instructions to fill the questionnaire:

1. Read detailed description of stressors before filling the questionnaire. Description is separately given below this questionnaire.
2. Fill only white cells and do not fill black and grey cells
3. Contextual Relationship = Leads to
4. What to enter in the white cells?

- Enter **V** when the row leads to the column
- Enter **A** when the column leads to the row
- Enter **O** when there is no relation between the row and the column
- Enter **X** when row and column lead to each other

Matrix indicating paired relationships

Stressors		1	2	3	4	5	6	7	8	9	10	11	12	13	14	15	16	17	18	19
**1**	High Job Demands																			
**2**	Low Job Control																			
**3**	Risk of COVID-19 Infection																			
**4**	Unavailability of proper PPEs																			
**5**	Inadequate Training																			
**6**	Work-Family Conflict																			
**7**	Anxious about Isolation/Quarantine																			
**8**	Subject to Violent Crimes																			
**9**	Feeling Frustrated and Powerless																			
**10**	Worried on Magnitude of Problem																			
**11**	Exhausting Shifts/hours without clear end																			
**12**	Shell Shock																			
**13**	Post-Traumatic Stress Disorder (PTSD)																			
**14**	Fear from Co-workers being COVID-19 Positive																			
**15**	No Mental Work Break																			
**16**	Lack of Proper Communication																			
**17**	Afraid of Spread of COVID-19 in Own Family																			
**18**	Sad for too many Dyeing Patients																			
**19**	Shortage of ventilators, beds, etc.																			

Annexure 3

Table A1: Iteration I

Code	Reachability Set	Antecedent Set	Intersection Set	Level
**1**	1,2,3,4,5,6,7,8,9,10,11,12,13,14,15,16,17,18,19	1,2,3,4,5,6,7,9,10,12,14,15,16,17,18,19	1,2,3,4,5,6,7,9,10,12,14,15,16,17,18,19	
**2**	1,2,3,4,5,6,7,8,9,10,11,12,13,14,15,17,18	1,2,3,4,5,6,7,9,10,11,12,13,14,15,16,17,18,19	1,2,3,4,5,6,7,9,10,11,12,13,14,15,17,18	
**3**	1,2,3,5,6,7,8,9,10,11,12,13,14,15,16,17,18,19	1,2,3,4,5,6,7,9,10,11,12,13,14,15,16,17,18,19	1,2,3,5,6,7,9,10,12,14,15,16,17,18,19	
**4**	1,2,3,4,5,6,7,8,9,10,11,12,13,14,15,16,17,18,19	1,2,4,5,6,7,9,10,13,14,15,16,17,18,19	1,2,4,5,6,7,9,10,13,14,15,16,17,18,19	
**5**	1,2,3,4,5,6,7,8,9,10,11,12,13,14,15,16,17,18,19	1,2,3,4,5,6,7,9,10,11,12,13,14,15,16,17,18,19	1,2,3,4,5,6,7,9,10,11,12,13,14,15,16,17,18,19	
**6**	1,2,3,4,5,6,7,8,9,10,11,12,13,14,15,17,18,19	1,2,3,4,5,6,7,9,10,13,14,15,16,17,18,19	1,2,3,4,5,6,7,9,10,13,14,15,17,18,19	
**7**	1,2,3,4,5,6,7,9,10,11,12,13,14,15,17,18	1,2,3,4,5,6,7,9,10,11,12,13,14,15,16,17,18,19	1,2,3,4,5,6,7,9,10,11,12,13,14,15,17,18	*I*
**8**	8	1,2,3,4,5,6,8,10,12,13,14,15,16,17,18,19	8	*I*
**9**	1,2,3,4,5,6,7,9,10,11,12,13,14,15,16,17,18,19	1,2,3,4,5,6,7,9,10,11,12,13,14,15,16,17,18,19	1,2,3,4,5,6,7,9,10,11,12,13,14,15,16,17,18,19	*I*
**10**	1,2,3,4,5,6,7,8,9,10,11,12,13,14,15,16,17,18,19	1,2,3,4,5,6,7,9,10,11,12,13,14,15,16,17,18,19	1,2,3,4,5,6,7,9,10,11,12,13,14,15,16,17,18,19	
**11**	2,3,5,7,9,10,11,12,13,14,15,17,18	1,2,3,4,5,6,7,9,10,11,12,13,14,15,16,17,18,19	2,3,5,7,9,10,11,12,13,14,15,17,18	*I*
**12**	1,2,3,5,7,8,9,10,11,12,13,14,15,17,18	1,2,3,4,5,6,7,9,10,11,12,13,14,15,16,17,18,19	1,2,3,5,7,9,10,11,12,13,14,15,17,18	
**13**	2,3,4,5,6,7,8,9,10,11,12,13,14,15,16,17,18,19	1,2,3,4,5,6,7,9,10,11,12,13,14,15,16,17,18,19	2,3,4,5,6,7,9,10,11,12,13,14,15,16,17,18,19	
**14**	1,2,3,4,5,6,7,8,9,10,11,12,13,14,15,16,17,18,19	1,2,3,4,5,6,7,9,10,11,12,13,14,15,16,17,18,19	1,2,3,4,5,6,7,9,10,11,12,13,14,15,16,17,18,19	
**15**	1,2,3,4,5,6,7,8,9,10,11,12,13,14,15,16,17,18,19	1,2,3,4,5,6,7,9,10,11,12,13,14,15,16,17,18,19	1,2,3,4,5,6,7,9,10,11,12,13,14,15,16,17,18,19	
**16**	1,2,3,4,5,6,7,8,9,10,11,12,13,14,15,16,17,18,19	1,3,4,5,9,10,13,14,15,16,18,19	1,3,4,5,9,10,13,14,15,16,18,19	
**17**	1,2,3,4,5,6,7,8,9,10,11,12,13,14,15,17,18,19	1,2,3,4,5,6,7,9,10,11,12,13,14,15,16,17,18,19	1,2,3,4,5,6,7,9,10,11,12,13,14,15,17,18,19	
**18**	1,2,3,4,5,6,7,8,9,10,11,12,13,14,15,16,17,18,19	1,2,3,4,5,6,7,9,10,11,12,13,14,15,16,17,18,19	1,2,3,4,5,6,7,9,10,11,12,13,14,15,16,17,18,19	
**19**	1,2,3,4,5,6,7,8,9,10,11,12,13,14,15,16,17,18,19	1,3,4,5,6,9,10,13,14,15,16,17,18,19	1,3,4,5,6,9,10,13,14,15,16,17,18,19	

Table A2: Iteration II

Code	Reachability Set	Antecedent Set	Intersection Set	Level
**1**	1,2,3,4,5,6,10,12,13,14,15,16,17,18,19	1,2,3,4,5,6,10,12,14,15,16,17,18,19	1,2,3,4,5,6,10,12,14,15,16,17,18,19	
**2**	1,2,3,4,5,6,10,12,13,14,15,17,18	1,2,3,4,5,6,10,12,13,14,15,16,17,18,19	1,2,3,4,5,6,10,12,13,14,15,17,18	*II*
**3**	1,2,3,5,6,10,12,13,14,15,16,17,18,19	1,2,3,4,5,6,10,12,13,14,15,16,17,18,19	1,2,3,5,6,10,14,15,16,17,18,19	
**4**	1,2,3,4,5,6,10,12,13,14,15,16,17,18,19	1,2,4,5,6,10,13,14,15,16,17,18,19	1,2,4,5,6,10,13,14,15,16,17,18,19	
**5**	1,2,3,4,5,6,10,12,13,14,15,16,17,18,19	1,2,3,4,5,6,10,12,13,14,15,16,17,18,19	1,2,3,4,5,6,10,12,13,14,15,16,17,18,19	*II*
**6**	1,2,3,4,5,6,10,12,13,14,15,17,18,19	1,2,3,4,5,6,10,13,14,15,16,17,18,19	1,2,3,4,5,6,10,13,14,15,17,18,19	
**10**	1,2,3,4,5,6,10,12,13,14,15,16,17,18,19	1,2,3,4,5,6,10,12,13,14,15,16,17,18,19	1,2,3,4,5,6,10,12,13,14,15,16,17,18,19	*II*
**12**	1,2,3,5,10,12,13,14,15,17,18	1,2,3,4,5,6,10,12,13,14,15,16,17,18,19	1,2,3,5,10,12,13,14,15,17,18	*II*
**13**	2,3,4,5,6,10,12,13,14,15,16,17,18,19	1,2,3,4,5,6,10,12,13,14,15,16,17,18,19	2,3,4,5,6,10,12,13,14,15,16,17,18,19	*II*
**14**	1,2,3,4,5,6,10,12,13,14,15,16,17,18,19	1,2,3,4,5,6,10,12,13,14,15,16,17,18,19	1,2,3,4,5,6,10,12,13,14,15,16,17,18,19	*II*
**15**	1,2,3,4,5,6,10,12,13,14,15,16,17,18,19	1,2,3,4,5,6,10,12,13,14,15,16,17,18,19	1,2,3,4,5,6,10,12,13,14,15,16,17,18,19	*II*
**16**	1,2,3,4,5,6,10,12,13,14,15,16,17,18,19	1,3,4,5,10,13,14,15,16,18,19	1,3,4,5,10,13,14,15,16,18,19	
**17**	1,2,3,4,5,6,10,12,13,14,15,17,18,19	1,2,3,4,5,6,10,12,13,14,15,16,17,18,19	1,2,3,4,5,6,10,12,13,14,15,17,18,19	*II*
**18**	1,2,3,4,5,6,10,12,13,14,15,16,17,18,19	1,2,3,4,5,6,10,12,13,14,15,16,17,18,19	1,2,3,4,5,6,10,12,13,14,15,16,17,18,19	*II*
**19**	1,2,3,4,5,6,10,12,13,14,15,16,17,18,19	1,3,4,5,6,10,13,14,15,16,17,18,19	1,3,4,5,6,10,13,14,15,16,17,18,19	

Table A3: Iteration III

Code	Reachability Set	Antecedent Set	Intersection Set	Level
**1**	1,3,4,6,16,19	1,3,4,6,16,19	1,3,4,6,16,19	*III*
**3**	1,3,6,16,19	1,3,4,6,16,19	1,3,6,16,19	*III*
**4**	1,3,4,6,16,19	1,4,6,16,19	1,4,6,16,19	
**6**	1,3,4,6,19	1,3,4,6,16,19	1,3,4,6,19	*III*
**16**	1,3,4,6,16,19	1,3,4,16,19	1,3,4,16,19	
**19**	1,3,4,6,16,19	1,3,4,6,16,19	1,3,4,6,16,19	*III*

Table A4: Iteration IV

Codes	Reachability Set	Antecedent Set	Intersection Set	Level
**4**	4,16	4,16	4,16	*IV*
**16**	4,16	4,16	4,16	*IV*

Table A5: Summary of Iterations

Code	Reachability Set	Antecedent Set	Intersection Set	Level
**7**	1,2,3,4,5,6,7,9,10,11,12,13,14,15,17,18	1,2,3,4,5,6,7,9,10,11,12,13,14,15,16,17,18,19	1,2,3,4,5,6,7,9,10,11,12,13,14,15,17,18	*I*
**8**	8	1,2,3,4,5,6,8,10,12,13,14,15,16,17,18,19	8	*I*
**9**	1,2,3,4,5,6,7,9,10,11,12,13,14,15,16,17,18,19	1,2,3,4,5,6,7,9,10,11,12,13,14,15,16,17,18,19	1,2,3,4,5,6,7,9,10,11,12,13,14,15,16,17,18,19	*I*
**11**	2,3,5,7,9,10,11,12,13,14,15,17,18	1,2,3,4,5,6,7,9,10,11,12,13,14,15,16,17,18,19	2,3,5,7,9,10,11,12,13,14,15,17,18	*I*
**2**	1,2,3,4,5,6,10,12,13,14,15,17,18	1,2,3,4,5,6,10,12,13,14,15,16,17,18,19	1,2,3,4,5,6,10,12,13,14,15,17,18	*II*
**5**	1,2,3,4,5,6,10,12,13,14,15,16,17,18,19	1,2,3,4,5,6,10,12,13,14,15,16,17,18,19	1,2,3,4,5,6,10,12,13,14,15,16,17,18,19	*II*
**10**	1,2,3,4,5,6,10,12,13,14,15,16,17,18,19	1,2,3,4,5,6,10,12,13,14,15,16,17,18,19	1,2,3,4,5,6,10,12,13,14,15,16,17,18,19	*II*
**12**	1,2,3,5,10,12,13,14,15,17,18	1,2,3,4,5,6,10,12,13,14,15,16,17,18,19	1,2,3,5,10,12,13,14,15,17,18	*II*
**13**	2,3,4,5,6,10,12,13,14,15,16,17,18,19	1,2,3,4,5,6,10,12,13,14,15,16,17,18,19	2,3,4,5,6,10,12,13,14,15,16,17,18,19	*II*
**14**	1,2,3,4,5,6,10,12,13,14,15,16,17,18,19	1,2,3,4,5,6,10,12,13,14,15,16,17,18,19	1,2,3,4,5,6,10,12,13,14,15,16,17,18,19	*II*
**15**	1,2,3,4,5,6,10,12,13,14,15,16,17,18,19	1,2,3,4,5,6,10,12,13,14,15,16,17,18,19	1,2,3,4,5,6,10,12,13,14,15,16,17,18,19	*II*
**17**	1,2,3,4,5,6,10,12,13,14,15,17,18,19	1,2,3,4,5,6,10,12,13,14,15,16,17,18,19	1,2,3,4,5,6,10,12,13,14,15,17,18,19	*II*
**18**	1,2,3,4,5,6,10,12,13,14,15,16,17,18,19	1,2,3,4,5,6,10,12,13,14,15,16,17,18,19	1,2,3,4,5,6,10,12,13,14,15,16,17,18,19	*II*
**1**	1,3,4,6,16,19	1,3,4,6,16,19	1,3,4,6,16,19	*III*
**3**	1,3,6,16,19	1,3,4,6,16,19	1,3,6,16,19	*III*
**6**	1,3,4,6,19	1,3,4,6,16,19	1,3,4,6,19	*III*
**19**	1,3,4,6,16,19	1,3,4,6,16,19	1,3,4,6,16,19	*III*
**4**	4,16	4,16	4,16	*IV*
**16**	4,16	4,16	4,16	*IV*

Table A6: Conical Matrix

	**7**	**8**	**9**	**11**	**2**	**5**	**10**	**12**	**13**	**14**	**15**	**17**	**18**	**1**	**3**	**6**	**19**	**4**	**16**
**7**	1	0	1	1*	1*	1*	1	1	1	1*	1*	1	1*	1*	1*	1*	0	1*	0
**8**	0	1	0	0	0	0	0	0	0	0	0	0	0	0	0	0	0	0	0
**9**	1*	0	1	1*	1*	1*	1	1*	1	1*	1*	1	1	1*	1	1*	1*	1	1*
**11**	1*	0	1*	1	1	1*	1*	1*	1	1*	1*	1*	1*	0	1	0	0	0	0
**2**	1	1*	1	1	1	1*	1	1*	1*	1	1*	1*	1*	1*	1	1*	0	1*	0
**5**	1	1	1	1	1	1	1	1*	1	1*	1	1	1	1	1*	1*	1*	1*	1*
**10**	1	1*	1	1	1	1*	1	1	1	1*	1*	1	1*	1	1*	1	1*	1*	1*
**12**	1*	1*	1*	1*	1	1	1*	1	1	1*	1*	1*	1*	1*	1*	0	0	0	0
**13**	1*	1*	1*	1*	1*	1*	1*	1*	1	1*	1	1	1	0	1	1*	1*	1*	1*
**14**	1	1	1	1*	1*	1	1	1	1	1	1	1	1	1	1	1*	1*	1	1*
**15**	1	1	1	1	1	1*	1*	1	1	1	1	1*	1	1*	1	1	1*	1	1*
**17**	1*	1*	1	1*	1	1*	1*	1*	1*	1	1*	1	1*	1*	1*	1	1*	1*	0
**18**	1	1*	1	1	1	1*	1*	1*	1	1*	1	1	1	1*	1	1	1	1*	1
**1**	1	1*	1	1*	1	1*	1*	1*	1	1*	1	1	1	1	1	1	1*	1*	1
**3**	1	1*	1*	1*	1*	1	1	1	1	1*	1*	1	1	1*	1	1*	1*	0	1*
**6**	1*	1	1*	1*	1*	1*	1	1*	1	1*	1	1*	1*	1*	1	1	1	1*	0
**19**	1	1*	1	1*	1	1	1	1*	1	1	1*	1*	1	1	1*	1*	1	1	1*
**4**	1	1*	1	1	1	1	1*	1*	1*	1*	1*	1	1*	1	1	1	1*	1	1*
**16**	1*	1	1	1*	1	1*	1*	1*	1*	1	1*	1*	1	1	1*	1*	1	1	1
